# Inhibitory Effects of a Reengineered Anthrax Toxin on Canine and Human Osteosarcoma Cells

**DOI:** 10.3390/toxins12100614

**Published:** 2020-09-24

**Authors:** Jonathan Mackowiak da Fonseca, Ivone Izabel Mackowiak da Fonseca, Marcia Kazumi Nagamine, Cristina de Oliveira Massoco, Adriana Tomoko Nishiya, Jerrold Michael Ward, Shihui Liu, Stephen Howard Leppla, Thomas Henrik Bugge, Maria Lucia Zaidan Dagli

**Affiliations:** 1 Department of Pathology, School of Veterinary Medicine and Animal Science, University of Sao Paulo, Sao Paulo 05508-270, SP, Brazil; jowfonseca@gmail.com (J.M.d.F.); ivonemfonseca@usp.br (I.I.M.d.F.); mknagamine@gmail.com (M.K.N.); cmassoco@usp.br (C.d.O.M.); adriananishiya@hotmail.com (A.T.N.); 2 Global Vet Pathology, Montgomery Village, MD 20886, USA; veterinarypathology@gmail.com; 3 Aging Institute and Division of Infectious Diseases, Department of Medicine, University of Pittsburg, Pittsburgh, PA 15261, USA; shl176@pitt.edu; 4 Microbial Pathogenesis Section, Laboratory of Parasitic Diseases, National Institute of Allergy and Infectious Diseases, National Institutes of Health, Bethesda, MD 20892, USA; sleppla@niaid.nih.gov; 5 Proteases & Tissue Remodeling Section, National Institute of Dental and Craniofacial Research, NIH, Bethesda, MD 20892, USA; tbugge@dir.nidcr.nih.gov

**Keywords:** toxin, canine osteosarcoma, *Bacillus anthracis*, anthrax, apoptosis

## Abstract

Canine and human osteosarcomas (OSA) share similarities. Novel therapies are necessary for these tumours. The *Bacillus anthracis* toxin was reengineered to target and kill cells with high expressions of matrix metalloproteinases (MMPs) and urokinase plasminogen activator (uPA). Since canine OSA express MMPs and uPA, we assessed whether the reengineered toxin could show efficacy against these tumours. Two OSA cell lines (canine D17 and human MG63) and a non-neoplastic canine osteoblastic cell line (COBS) were used. Cells were treated with different concentrations of the reengineered anthrax toxin and cell viability was quantified using MTT assay. The cell cycle, apoptosis, and necrosis were analysed by flow cytometry. The wound-healing assay was performed to quantify the migration capacity of treated cells. D17 and MG63 cells had significantly decreased viability after 24 h of treatment. Cell cycle analysis revealed that OSA cells underwent apoptosis when treated with the toxin, whereas COBS cells arrested in the G1 phase. The wound-healing assay showed that D17 and MG63 cells had a significantly reduced migration capacity after treatment. These results point for the first time towards the in vitro inhibitory effects of the reengineered anthrax toxin on OSA cells; this reengineered toxin could be further tested as a new therapy for OSA.

## 1. Introduction

Canine spontaneous tumours are considered good models for human disease due to their similar morphology and behavior. Canine tumours may represent a better alternative to rodent tumours for studying cancer biology and therapy [1,2].

Osteosarcoma (OSA) is the most common primary malignant bone tumour in dogs [3,4,5], being characterised by a high metastatic potential and poor prognosis [1,6,7]. Most dogs that develop canine OSA die because of lung metastases. Further, the recurrence rate is high and the median survival time ranges from 3 months to 1 year [8,9,10]. Therefore, new treatments are necessary for canine osteosarcomas, and targeted therapies could be adequate options.

In the search for new therapies for cancer, many natural compounds can be found that have the ability to kill cells. The *Bacillus anthracis* toxin is one of these compounds. However, due to its highly toxic effect on normal cells, the natural anthrax toxin would not be suitable for treating cancers without causing serious adverse effects.

The natural *Bacillus anthracis* toxin is composed of three parts: the oedema factor (EF), the lethal factor (LF), and the protective antigen (PA). The PA combined with the LF or EF is toxic to cells and may result in death in animals [11,12]. An alternative effector component, FP59, consists of the N-terminal 254 amino acids of LF fused to the catalytic domain of *Pseudomonas* exotoxin A. FP59 kills cells by inhibiting protein synthesis. The *Bacillus anthracis* toxin has been modified to specifically target tumour cells that express matrix metalloproteinases (MMPs) and the urokinase plasminogen activator (uPA) [13,14,15,16]. The reengineered toxin consists of the PA variants PA-U2-R200A and PA-L1-I210A, which cause cell death by disruption of the MAP kinase (MAPK) signalling pathway when combined with the LF.

The modified anthrax toxin is cytotoxic to xenografted human melanomas and carcinomas [17,18]. Recently, our group has shown the efficacy of the reengineered anthrax toxin in canine oral mucosal melanomas, where it resulted in decreased tumour growth and stable disease. In addition, tumours had decreased bleeding, and tumour cells and intratumoral endothelial cells were necrotic [19]. Although the reengineered *Bacillus anthracis* toxin has shown anticancer properties in carcinomas and melanomas, its effects have not been tested in mesenchymal tumours yet. Since osteosarcomas are so prevalent and aggressive in dogs and there are only a few effective treatments, we aimed to investigate if the reengineered anthrax toxin could exert any inhibitory effects on canine osteosarcoma cells in comparison to human osteosarcoma cells. Based on the in vitro inhibitory effects of this reengineered toxin on canine osteosarcoma cells, further studies will be proposed to investigate if it could be used as a novel therapy for osteosarcomas in vivo.

## 2. Results

### 2.1. Canine OSA Tissue Has High Expression of uPA and MMPs

Since it is known that uPA and MMPs are overexpressed in a variety of tumour cells and are rarely present in normal cells [20], we first evaluated their expression in a tissue microarray (TMA) containing 22 canine OSA samples. All of the samples were positive for MMP-2, MT1-MMP, uPA H140, and TIMP 2. These results led us to test the effects of reengineered anthrax toxins on canine OSA cells. Since canine tumours in dogs are considered good models for human cancers, we included both dog and human OSA cells in this study. In addition, a normal canine cell osteoblastic cell line, COBS, was also used in order to test the effect of the anthrax toxin on a non-neoplastic cell line.

### 2.2. OSA Cells Express Tumour Markers

**Figure 1:**
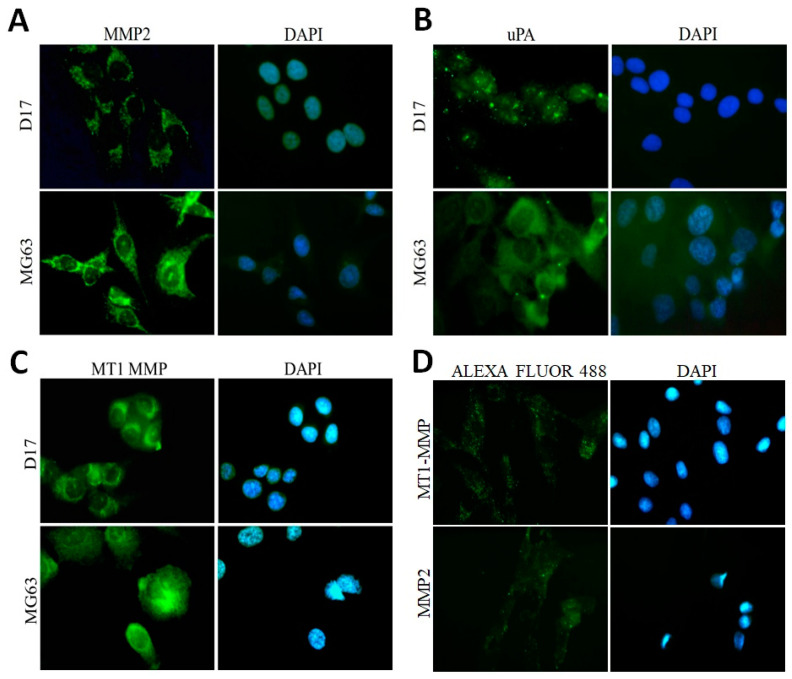
Photomicrograph representation of the expression of different markers in canine (D17) and human (MG63) osteosarcoma cell lines and canine osteoblastic (COBS) cell line, as tested by immunofluorescence. (**A**) Metalloproteinase 2 (MMP2) expression in canine (D17) and human (MG63) osteosarcoma cell lines. (**B**) The urokinase plasminogen activator (uPA)expression in canine (D17) and human (MG63) osteosarcoma cell lines. (**C**) Membrane-type 1 matrix metalloproteinase MT1-MMP expression in canine (D17) and human (MG63) osteosarcoma cell lines. (**D**) Membrane-type 1 matrix metalloproteinase (MT1-MMP) and Metalloproteinase 2 (MMP2) expression in Canine Osteoblast Spitz (COBS) cell line. Note: Alexa Fluor 488: green; DAPI: blue. Original magnification 40×.

We performed immunofluorescence assays to check the basal expression patterns of tumour markers in canine (D17) and human (MG63) OSA cell lines and a non-neoplastic canine osteoblastic cell line (COBS). The expression of MMP 2 (Figure 1A), uPA (Figure 1B), and MT1-MMP (Figure 1C) has been observed in both canine and human OSA cell lines. MT1-MMP and MMP2 expressions were also seen at lower intensities in COBS cell line (Figure 1D).

Regarding the immunoexpression of uPA (Figure 1B), it is possible to see cytoplasm positivity in both MG63 and D17, but also dot immunostainings in both cells lines, which we considered as indicator of the positivity of uPA in cell membranes. Although no quantification was performed, apparently D17 has more uPA immunostaining dots in the membrane than MG63.

The non-neoplastic canine osteoblastic cell line (COBS) was established in our laboratory after isolation of cells from a fragment of a dog’s bone fracture. This cell line was previously validated in our lab to express bone markers and has low expression of MMP2 and MTI-MMP [21].

### 2.3. The Reengineered Anthrax Toxin Is More Cytotoxic to OSA Cells Than to COBS Cells

LF and FP59 were applied to canine and human osteosarcoma cells, and it was observed that neither LF or FP59 alone was cytotoxic in all tested cells. Within a certain dose range, the cell viability was comparable to that of untreated cells, confirming that the toxicity was dependent on the other component of the toxin, the native PA.

**Figure 2:**
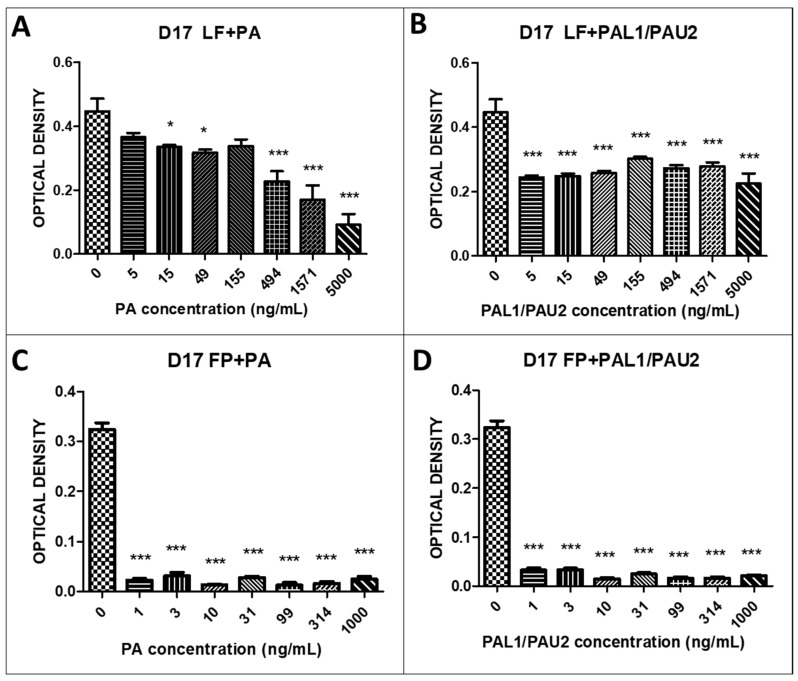
Response of the canine osteosarcoma cell line (D17) to the reengineered anthrax toxin. (**A**) D17 cells treated with LF + PA at differing PA concentrations; (**B**) D17 cells treated with LF + PAL1/PAU2 at differing PA concentrations. (**C**) D17 cells treated with FP59 + PA at differing PA concentrations. (**D**) D17 cells treated with FP59 + PAL1/PAU2 at differing PA concentrations. Optical density was compared to that of untreated cells (only LF or FP59). Note: * *p* < 0.05; *** *p* < 0.001. LF: lethal factor; PA: protective antigen; PAL1: PA-L1-l210; PAU2: PA-U2-R200A; FP59: the A exotoxin of *Pseudomonas aeruginosa* combined with the LF.

We also examined the reengineered anthrax toxin in OSA cells. LF plus PA at all concentrations (15, 49, 494, 1571, and 5000 ng/mL PA) caused significant cytotoxicity after 24 h in the canine OSA cell line D17 when compared to untreated cells (Figure 2A). Combination of LF with PA-U2-R200A and PA-L1-I210A also proved to be significantly cytotoxic at all PA concentrations (5, 15, 49, 155, 494, 1571, and 5000 ng/mL PA) in D17 cells (Figure 2B). When we analysed the combinations of FP59 and PA and FP59 and PA-U2-R200A and PA-L1-I210A in D17 cells, we observed statistically significant cytotoxicity at all applied concentrations (1, 3, 10, 31, 99, 314, and 1000 ng/mL PA) when compared to untreated cells (Figure 2C,D).

**Figure 3:**
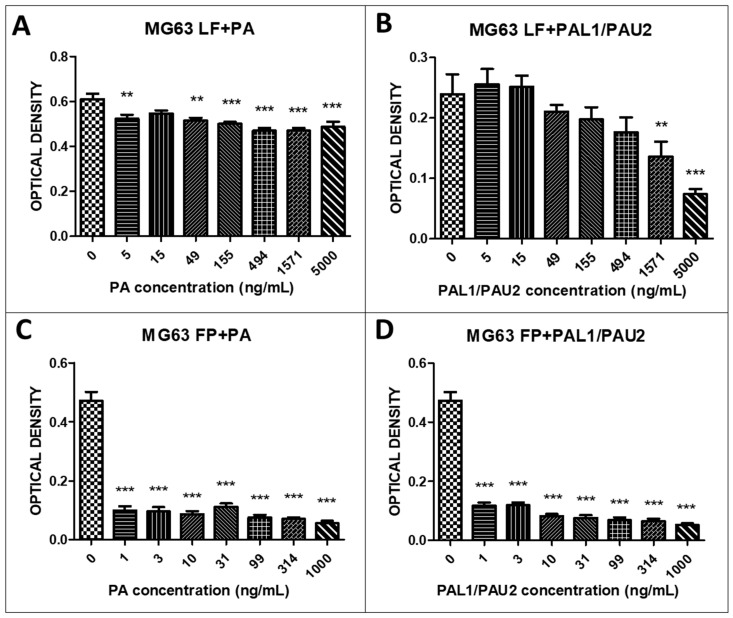
Response of the human osteosarcoma cell line (MG63) to the reengineered anthrax toxin. (**A**) MG63 cells treated with LF + PA at differing PA concentrations. (**B**) MG63 cells treated with LF + PAL1/PAU2 at differing PA concentrations. (**C**) MG63 cells treated with FP59 + PA at differing PA concentrations. (**D**) MG63 cells treated with FP59 + PAL1/PAU2 at differing PA concentrations. Optical density was compared to that of untreated cells (only LF or FP59). Note: ** *p* < 0.01; *** *p* < 0.001. LF: lethal factor; PA: protective antigen; PAL1: PA-L1-l210; PAU2: PA-U2-R200A; FP59: the A exotoxin of *Pseudomonas aeruginosa* combined with the LF.

We further examined the reengineered anthrax toxin in human OSA cell line MG63. The combination of LF and PA at 5, 49, 155, 494, 1571, and 5000 ng/mL PA was significantly cytotoxic in the human OSA cell line MG63 compared to untreated cells (Figure 3A). The combination of LF with PA-U2-R200A and PA-L1-I210A was also significantly cytotoxic; there was a decrease in the optical density at the two highest PA concentrations (1571 and 5000 ng/mL PA) (Figure 3B). When we analysed the combinations of FP59 and PA and FP59 and PA-U2-R200A/PA-L1-I210A in MG63 cells, we observed statistically significant cytotoxicity at all analysed concentrations (1, 3, 10, 31, 99, 314, and 1000 ng/mL PA) (Figure 3C,D).

**Figure 4:**
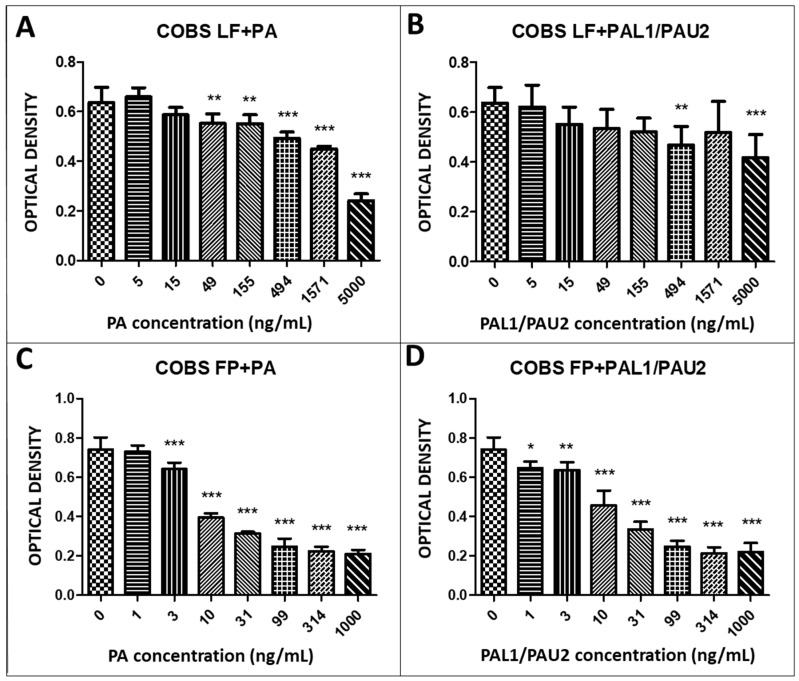
Response of the non-neoplastic canine osteoblastic cell line (COBS) to the reengineered anthrax toxin. (**A**) COBS cells treated with LF + PA at differing PA concentrations. (**B**) COBS cells treated with LF + PAL1/PAU2 at differing PA concentrations. (**C**) COBS cells treated with FP59 + PA at differing PA concentrations. (**D**) COBS cells treated with FP59 + PAL1/PAU2 at differing PA concentrations. Optical density was compared to that of untreated cells (only LF or FP59). Note: * *p* < 0.05; ** *p* < 0.01; *** *p* < 0.001. LF: lethal factor; PA: protective antigen; PAL1: PA-L1-l210; PAU2: PA-U2-R200A; FP59: the A exotoxin of *Pseudomonas aeruginosa* combined with the LF.

We next assessed the cytotoxicity of the reengineered toxin with a fixed concentration of LF and different concentrations of PA. After 24 h of treatment with the reengineered anthrax toxin with 49, 155, 494, 1571, and 5000 ng/mL PA, we observed a significant decrease in COBS cell viability compared to untreated cells (0 ng/mL PA) (Figure 4A). Combinations of LF with PA-U2-R200A and PA-L1-I210A at concentrations of 494 and 5000 ng/mL, respectively, were also significantly cytotoxic (Figure 4B). We also analysed the effects of FP59 plus PA in COBS cells. We observed statistically significant cytotoxicity at PA concentrations of 3, 10, 31, 99, 314, and 1000 ng/mL (Figure 4C). Similar to what we observed with the LF, the combination of FP59 and PA-U2-R200A/PA-L1-I210A caused significant cytotoxicity at all applied concentrations (1, 3, 10, 31, 99, 314, and 1000 ng/mL PA) (Figure 4D).

**Figure 5:**
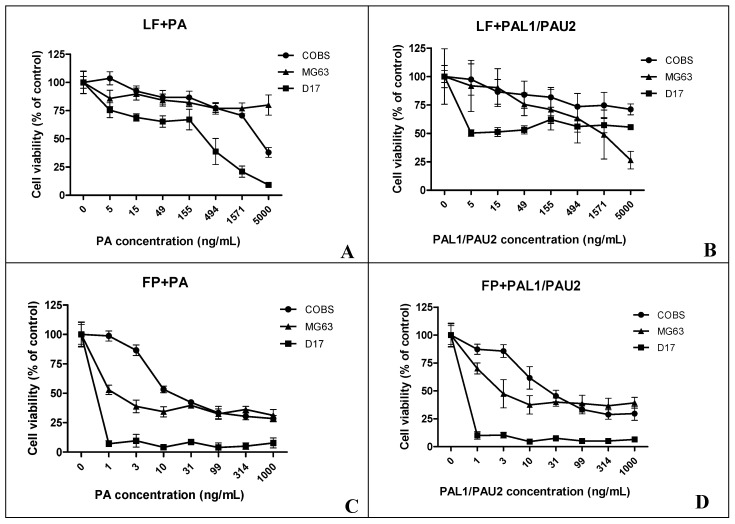
Graphs showing the cell viability of canine and human osteosarcoma cell lines (D17 and MG63, respectively) and canine osteoblast cell line (COBS) after treatment with reengineered anthrax toxins: (**A**) LF + PA; (**B**) LF + PAL1/PAU2; (**C**) FP + PA; (**D**) FP + PAL1/PAU2. The IC50 values are shown in Table 1.

The cell viability upon treating D17, MG63, and COBS with the toxin parts was analysed in accordance to the IC50 (half minimal inhibitory concentration). As can be seen in Table 1, OSA cell lineages MG63 and D17 have IC50 values lower than the normal cell lineage of COBS, except for the value found for the LF + PA treatment, in which MG63 shows a much higher IC50 value. Cell line D17 showed the highest sensitivity to treatment with reengineered anthrax toxins (Figure 5).

The D17, MG63, and COBS values of viability were normalized and are presented in Figure 5.

In Figure 5A, we have the exposure of cells to the LF + PA fraction of the toxin, which is much more toxic to cell lines. We observed that the human osteosarcoma cell line (MG63) was resistant to the toxin in all concentrations in this treatment (LF + PA), except in the highest concentration (5000 ng/mL). The normal canine osteoblastic lineage (COBS) remained constant for almost all treatment concentrations, thus showing resistance to the toxin at different concentrations. The canine osteosarcoma cell line (D17), on the other hand, showed a decline in cell viability from the concentration of 155 ng/mL onwards, showing itself to be the most sensitive strain to treatment with the toxin LF + PA.

Figure 5B shows the exposure of the cells to the LF + PAL/PAU fraction of the reengineered toxin, a therapeutic portion, which was less toxic to the tested cell lines. We observed that the human osteosarcoma cell line (MG63) was sensitive to treatment with the fraction (LF + PAL/PAU) of the reengineered toxin from the concentration of 155 ng/mL onwards. In the normal canine osteoblastic strain (COBS), we observed a small decline in cell viability, but this viability was stable even during exposure to the highest concentrations of the toxin (LF + PAL/PAU). The canine osteosarcoma cell line (D17) was more sensitive in the first concentrations of the toxin LF + PAL/PAU (5, 15 and 49 ng/mL), but from the concentration 155 ng/mL on wards it showed stability in terms of cell viability.

Figure 5C shows the exposure of cells to the FP + PA fraction of the toxin. This modified FP fraction is 50 times more potent than wild LF, which is used to detect the presence of toxin receptors in the strains analyzed. We observed that the human osteosarcoma cell line (MG63) and the canine osteosarcoma cell line (D17) showed great sensitivity from the concentration of 1 ng/mL onwards when treated with the FP + PA fraction of the toxin, thus demonstrating the presence of receptors that respond to the toxin. The same did not happen with normal canine osteoblastic (COBS), which was not as sensitive to this fraction with the lowest concentrations of the toxin; only after the concentration of 10 ng/mL did it begin to decrease its viability.

In Figure 5D, we observe the exposure of cells to the FP + PAL/PAU fraction of the reengineered toxin. This association showed practically the same results as observed with the FP + PA portion. The canine osteosarcoma cell line (D17) was more sensitive than the other two cell lines, MG63 and COBS.

### 2.4. The Reengineered Anthrax Toxin Causes Cell Death in OSA Cells

**Figure 6:**
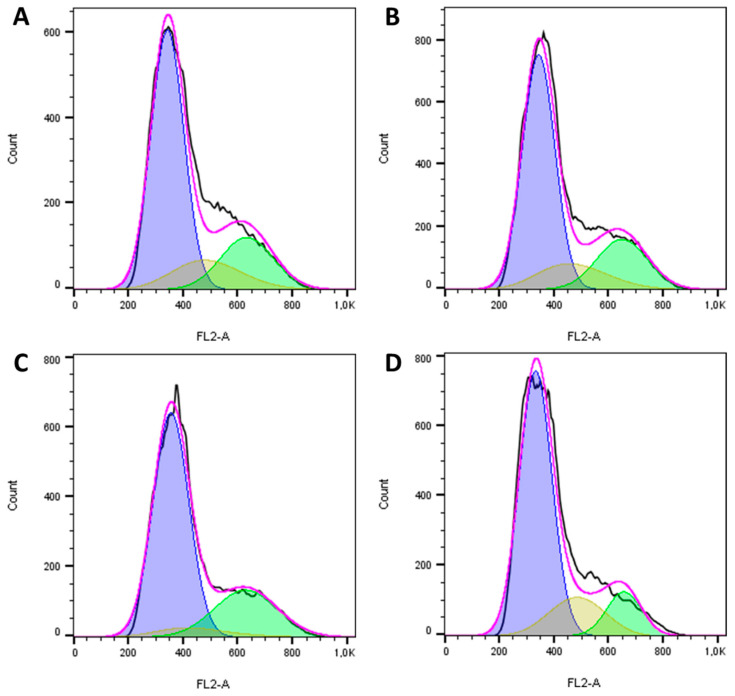
Cell cycle histograms of the canine osteosarcoma cell line D17 after 24 h of treatment with the reengineered anthrax toxin from *Bacillus anthracis*: (**A**) LF only; (**B**) LF + PAL1/PAU2 50 ng/mL; (**C**) LF + PAL1/PAU2 500 ng/mL; (**D**) LF + PAL1/PAU2 5000 ng/mL. LF: lethal factor; PAL1: PA-L1-l210; PAU2: PA-U2-R200A. The black line represents a cell population histogram in respect to iodide propide fluorescence, while the pink line represents the univariate cell cycle model created by FlowJo software to de-convolute the populations in order to assign percentage values to each population, since adjacent populations overlap each other, resulting in the purple, yellow, and green areas, which represent G0/G1, S, and G2/M phases, respectively.

**Figure 7:**
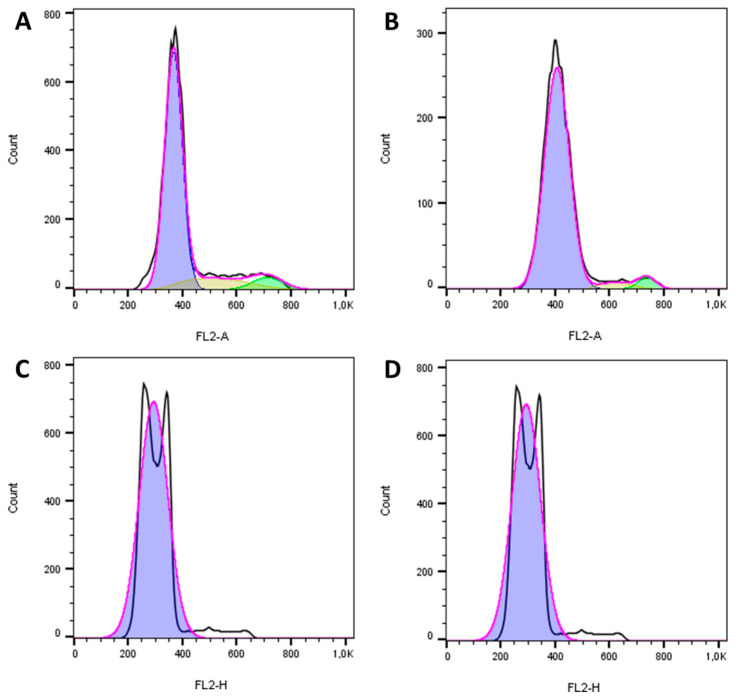
Cell cycle histograms of the human osteosarcoma cell line MG63 after 24 h of treatment with the reengineered anthrax toxin from *Bacillus anthracis*: (**A**) LF only; (**B**) LF + PAL1/PAU2 50 ng/mL; (**C**) LF + PAL1/PAU2 500 ng/mL; (**D**) LF + PAL1/PAU2 5000 ng/mL. LF: lethal factor; PAL1: PA-L1-l210; PAU2: PA-U2-R200A.

**Figure 8:**
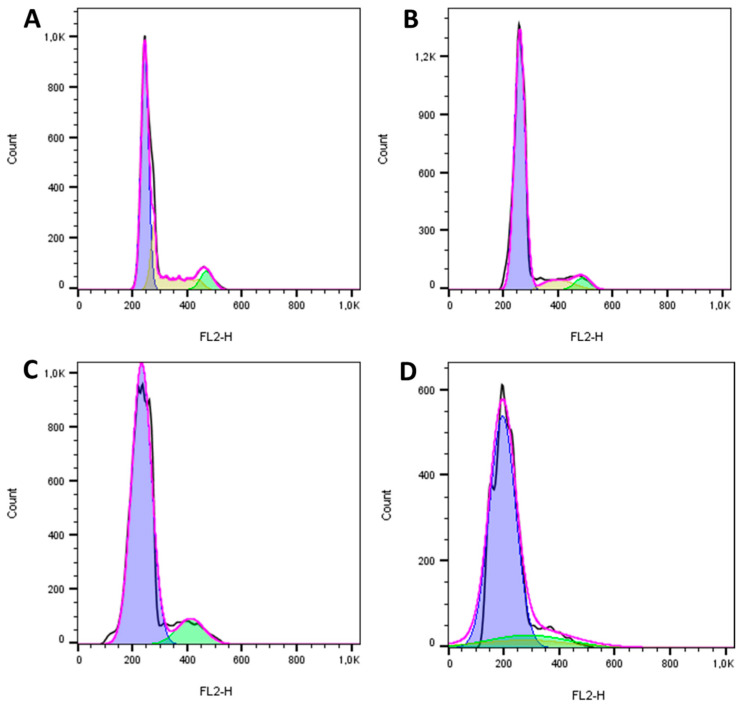
Cell cycle histograms of the non-neoplastic COBS cell line after 24 h of treatment with the reengineered anthrax toxin from *Bacillus anthracis*: (**A**) LF only; (**B**) LF + PAL1/PAU2 50 ng/mL: (**C**) LF + PAL1/PAU2 500 ng/mL; (**D**) LF + PAL1/PAU2 5000 ng/mL. LF: lethal factor; PAL1: PA-L1-l210; PAU2: PA-U2-R200A.

Cell cycle analysis indicated that the reengineered anthrax toxin caused apoptosis in the canine OSA cell line D17 (Figure 6) and in the human OSA cell line MG63 (Figure 7), as can be seen in the representative histograms, which revealed the fractional DNA content (sub-G1peak) and S-phase arrest. In contrast, the reengineered toxin caused arrest in the G1 phase in the non-neoplastic canine osteoblastic COBS cell line when analyzing the cell distribution at each phase of the cell cycle, even at the highest concentration (Figure 8).

### 2.5. The Reengineered Anthrax Toxin Decreased Migration Capacity in OSA Cells

**Figure 9:**
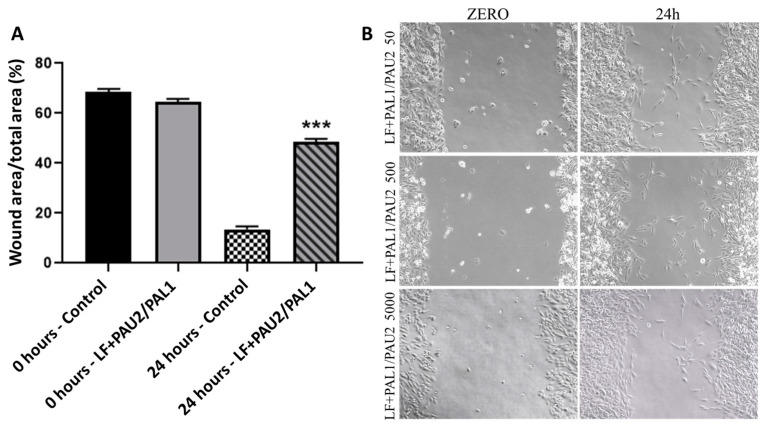
Relative width of the lesion in the wound-healing assay in the canine osteosarcoma cell line D17 with and without treatment of LF + PAL1/PAU2 at a concentration of 5000 ng/mL PA. (**A**) D17 cells treated with LF + PAL1/PAU2 at 5000 ng/mL PA. (**B**) Representative images of the wound-healing assay in the canine osteosarcoma cell line D17. Note: *** *p* < 0.001 in relation to the control at 24 h. LF: lethal factor; PAL1: PA-L1-l210; PAU2: PA-U2-R200A.

The wound-healing assay was performed to evaluate the cell migration capacity after treatment with the reengineered anthrax toxin. After 24 h of treatment with only the LF portion of the toxin, the canine OSA cell line D17 had good capacity to grow and migrate into the space left by the wound, partially closing it. This response was similar to that observed in control cells (Figure 8). In contrast, after 24 h of treatment with LF + PA-U2-R200A/PA-L1-I210A, D17 cells lost the ability to migrate into the space left by the wound (Figure 9).

**Figure 10:**
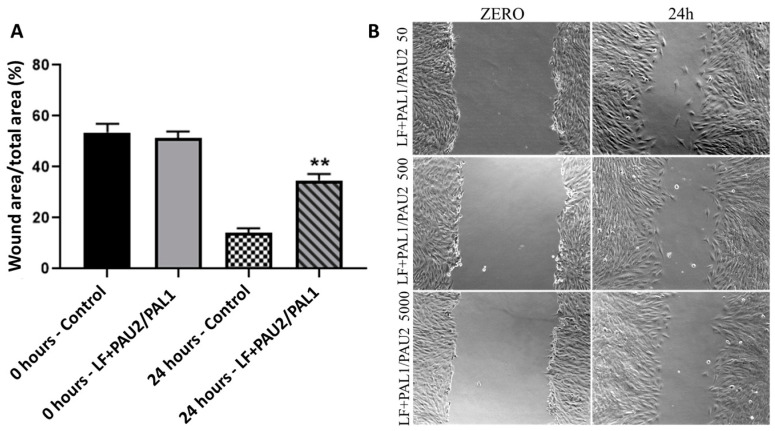
Relative area of the lesion in the wound-healing assay in the human osteosarcoma cell line MG63 with and without LF + PAL1/PAU2 at 5000 ng/mL PA. (**A**) MG63 cells treated with LF + PAL1/PAU2 at 5000 ng/mL PA. (**B**) Representative images of the wound-healing assay in the human osteosarcoma cell line MG63. Note: ** *p* < 0.01 in relation to the control at 24 h. LF: lethal factor; PAL1: PA-L1-l210; PAU2: PA-U2-R200A.

The human OSA cell line MG63 had good migratory capacity after 24 h of treatment with only the LF portion of the toxin, partially closing the wound. This was similar to that observed in the control cells (Figure 10). In contrast, after 24 h of treatment with LF + PA-U2-R200A/PA-L1-I210A, MG63 cells had significantly lost the ability to migrate into the space left by the wound (Figure 9).

## 3. Discussion

Osteosarcomas are aggressive neoplasms in both dogs and humans. In this study, we aimed to test a possible novel targeted therapy for these tumors in vitro. The *Bacillus anthracis* toxin is naturally toxic and has been modified to target some components (uPA and metalloproteinases) that are often highly expressed in cancer cells.

New selective therapies have shown encouraging results in the treatment of different types of neoplasms. One such possible selective therapy uses the genetically reengineered *B. anthracis* toxin. The *B. anthracis* toxin has three subunits: an EF, a PA, and an LF. The furin proteases cleave the PA at the cell membrane, forming an active heptamer (PA63) bound to the *B. anthracis* toxin receptor (TEM8 or CMG2). The channel formed by this association allows EF and LF to translocate into the cytosol, causing cell death either by increasing intracellular cAMP levels [11,15,22,23,24,25] or blocking MAPK signaling [3,26,27]. Metalloproteinases and urokinase are mainly expressed in neoplastic cells [17,28,29]. Therefore, the *B. anthracis* toxin was modified (mutated PA) so as to be activated by uPA and MMPs. Liu et al. synthesized the PA variants PA-L1-I210A and PA-U2-R200A, which can only be cleaved by MMP and uPA, respectively [12,16,22,30]. This modification allowed the toxin to act selectively in cells that express both proteases at their outer membranes, and it has diminished off-target cytotoxicity when compared to the native *B. anthracis* toxin [16].

Before testing whether this modified toxin would be effective in canine and human OSA cell lines, we first examined the MMP, uPA, and MT1-MMP expression in canine (D17) and human (MG63) OSA by immunofluorescence assay. Immunofluorescence in D17 and MG63 OSA cell lines indicated the expression of MMP2, MT1-MMP, and uPA. Although we did not comparatively quantify the expression of uPA in canine and human OSA cells, apparently the canine OSA has high numbers of immunoflurescent dots, suggesting a higher expression of uPA in these canine cancer cells. The significance of this is unknown. The uPA expression has scarcely been studied in canine tumors, either through immunohistochemistry of selective tumors [31,32,33] or its serum levels [34]. Its functions and importance in cancer, however, seems to be similar, at least in canine mammary tumors [35], and correlated to more aggressive and metastatic tumors.

The expression of uPA and MMP2 suggested that these OSA cell lines could be sensitive to the reengineered *B. anthracis* toxin, as it was modified to have a selective cytotoxic action. Therefore, we used these lines for toxin cytotoxicity tests. In addition, in our experiments we also used the non-neoplastic canine osteoblastic cell line (COBS). This line has low expression of MMP2 and MMP-MT1 [21]. We must state here that no human osteoblastic cell line was available for our experiments, and that comparisons between canine and human osteosarcoma cell lines and normal canine cell lines were not performed. Every cell line tested had its own controls, represented by treatments with other parts of the reengineered toxin. Our results corroborate those of previous studies [36], which showed a significantly higher expression of these markers in tumour tissues than in normal tissues. These data suggested that the reengineered toxin could be cytotoxic in OSA cell lines, since the action of the toxin is dependent on the presence of proteases, such as MMPs and uPAs. This toxin would also be selective in cancer cells, since normal tissues have low or no expression of these markers.

We observed that the LF and FP59 portions of the toxin alone were not cytotoxic in any of the cell lines, confirming that the reengineered *B. anthracis* toxin was dependent on the association with PA for cytotoxicity. In the D17 canine OSA cell line, the combination of LF and PA at all PA concentrations (5, 15, 49, 155, 494, 1571, and 5000 ng/mL) was significantly cytotoxic. The combination of LF and PAL1/PAU2, the modified toxin, also proved to be significantly cytotoxic at all concentrations of PA. We also found that combinations of FP59 and PA and FP59 and PA-U2-R200A/PA-L1-I210A were more cytotoxic in D17 cells than the LP combinations. This higher mortality caused by FP59 in relation to LF was expected, since FP59 is 50 times more potent than LF. We observed similar results in the MG63 human OSA cell line. The combination of LF and PA was cytotoxic at 5, 49, 155, 494, 1571, and 5000 ng/mL PA. The combinations of LF and PA-U2-R200A/PA-L1-I210A was also cytotoxic at the two highest PA concentrations (1571 and 5000 ng/mL). As with the D17 cells, the combinations of FP59 and PA and FP59 and PA-U2-R200A/PA-L1-I210A in MG63 cells had even higher cytotoxicity at all PA concentrations examined (1, 3, 10, 31, 99, 314, and 1000 ng/mL). These results indicated that MG63 and D17 cells have receptors for urokinase and metalloproteinases, allowing the *B. anthracis* toxins to internalise. Cell cycle analysis indicated that this cytotoxicity was due to apoptosis.

The non-neoplastic canine osteoblastic cell line (COBS) showed sensitivity to the reengineered *B. anthracis* toxin only at two of the three highest concentrations (494 and 5000 ng/mL) of the therapeutic toxin (LF + PA-U2-R200A/PA-L1-I210A). This suggests that this normal cell line has receptors for the *B. anthracis* toxin (TEM8 or CMG2). However, the expression levels of MMP2 and MT1-MMP are low, which explains the lower sensitivity compared with the two OSA cell lines. In addition, cell cycle analysis showed that the reengineered toxin did not induce apoptosis in COBS cells, unlike in the two OSA cell lines, but only arrested the cells in the G1 phase.

We can conclude that canine and human OSA cells were sensitive to treatment with reengineered *B. anthracis* toxin, which caused cell death. Further, the LF toxin with PA-U2-R200A/PA-L1-I210A was less aggressive in the normal osteoblastic cell line; therefore, this toxin is selective for cancer cells and has the best therapeutic potential. This cytotoxicity of *B. anthrax* toxin is due to the action of the LF, which is a zinc metalloproteinase that cleaves Nlrp1 [37] and MAPKK-activated protein kinase in its N-terminal region [26]. The cleavage of Nlrp1 by LF causes activation of inflammasomes, release of IL-1β, and pyroptosis of macrophages in mice and rats [32]. MAPKKK cleavage interrupts several signalling pathways, including the ERK1/2, JNK/SAPK, and p38 pathways. These pathways have important cellular functions, such as proliferation and regulation, but they also regulate immune modulation and survival against insults. Our cell cycle analysis indicated that the cell death observed with reengineered toxin treatment was due to apoptosis. These results corroborate those of previous studies, which showed that *B. anthracis* toxin interfered with protein synthesis and caused DNA damage, leading to apoptosis [11].

Cell migration analysis was performed to observe the effect of the toxin on migration capacity. In the two OSA cell lines, the migration capacity was compromised after treatment with the therapeutic reengineered *B. anthracis* toxin at all concentrations. These effects were not described before in studies involving the reengineered *B. anthracis* toxin and cancer cells, and may indicate that the toxin affects the capacity of cancer cell migration in vivo as well. Since invasion and migration are initial steps towards metastasis, it is possible that the toxin will not only inhibit the local tumour growth but also may affect its metastatic potential.

In this study, we show that the reengineered anthrax toxin determines the inhibitory effects on canine and human osteosarcoma cells. A series of studies have shown similarities between canine and human osteosarcomas [38,39,40] regarding the morphology and molecular alterations. In particular, canine osteosarcomas can be models for pediatric osteosarcomas [41]. Therefore, new drugs that can inhibit canine tumors, including osteosarcomas, can be tested in humans, eventually children, and represent novel therapies for these aggressive diseases.

## 4. Conclusions

The reengineered anthrax toxin is a promising potential treatment for canine OSA and possibly for other solid tumours that express metalloproteinases and the urokinase plasminogen activator, uPA. The potential to decrease cancer cell migration is indicative that this anthrax toxin has the potential to inhibit the initial steps for metastasis. Therefore, it deserves further research and clinical tests in order to show its safety and efficacy in vivo, so that it can be used to treat canine (and eventually human) osteosarcomas.

## 5. Materials and Methods

### 5.1. Cell Lines

The study was performed in accordance with protocols approved by the Committee on Ethics on the Use of Animals of the School of Veterinary Medicine and Animal Science of the University of São Paulo (FMVZ-USP), Brazil, process number 5645170918, 17 April 2019.

**Figure 11:**
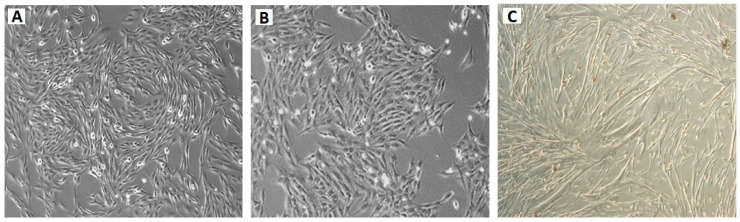
Photomicrograph representative of canine (D17) and human (MG63) osteosarcoma cell lines and canine osteoblastic cell line (COBS) in culture: (**A**) canine osteosarcoma cell line (D17); (**B**) human osteosarcoma cell line (MG63); (**C**) canine osteoblastic cell line (COBS). Original magnification 40×.

Two different OSA cell lines were used—a commercial canine OSA cell line, D17 (Figure 11A), and a commercial human OSA cell line, MG63 (Figure 11B). The canine OSA strain D17 was purchased from the American Type Culture Collection (ATCC) (Manassas, VA, USA) (ATCC^®^ CCL-183™). This cell line comes from a female dog, poodle breed, 11 years old, and was purchased at passage 227. The cells are adherent and have epithelial morphology. The human OSA cell line MG63 was also purchased from the ATCC (code: ATCC^®^ CRL-1427™). This cell line comes from a 14-year-old male. The cells are adherent and have fibroblastic morphology.

We previously established a non-neoplastic canine osteoblastic cell line (COBS) (Figure 11C) from a bone callus scrape from a fracture in the tibia of a female dog, German Spitz dwarf breed, aged 10 months, which we called the COBS cell line. These cells were submitted for karyotyping and have 78 chromosomes, the normal number for canines. They are positive for osteonectin, alkaline phosphatase, osteopontin, and secreted protein, and are acidic and rich in cysteine [21].

The OSA cell lines were cultured in DMEM with 10% foetal bovine serum, 100 μg/mL penicillin and 100 μg/mL streptomycin (Gibco™ Penicillin-Streptomycin (10,000 U/mL, Carlsbad, CA, USA)) in an incubator at 5% CO_2_ and in a humidified atmosphere at 37 °C. The COBS cell line was isolated and cultivated in a complete medium specific for canine osteoblast growth (canine osteoblast growth medium, code: CN417500, Sigma-Aldrich, Saint-Louis, MO, USA).

### 5.2. Reengineered Anthrax Toxin

The reengineered anthrax toxin was kindly supplied by Drs. Leppla, Bugge, and Liu [11,17,18] from the Laboratory of Parasitic Diseases of the National Institute of Allergy and Infectious Diseases of the National Institutes of Health in Bethesda, MD, USA. The importation of the toxin was approved by the National Sanitary Surveillance Agency of the Brazilian Ministry of Health to be acquired by the Laboratory of Experimental Oncology of the Department of Pathology at the School of Veterinary Medicine and animal Science of the University of Sao Paulo, USP, and was kept in a freezer at −80 °C.

The following parts of the anthrax toxin were supplied:

- The native protective antigen (PA);
- The reengineered protective antigen: PA-U2-R200A + PA-L1-I210A;
- The lethal factor (LF)
- The catalytic domain of *Pseudomonas aeruginosa* exotoxin A combined with the N-terminal domain of LF (FP59).

### 5.3. Immunofluorescence

For immunofluorescence, the OSA cell lines and the COBS cell line were grown on cover slips in 6-well plates. After 2 days, the medium was removed and the cells were washed with PBS and fixed with 70% ethanol for 20 min at −20 °C. The cells were subjected to antigenic exposure with EDTA pH 9.0 buffer heated to 90 °C for 20 min. After antigenic exposure, the coverslips were washed with 1 × PBS plus Triton X-100. Then, the coverslips were incubated in a humid chamber overnight at 4 °C with the antibodies, as shown in Table 2. The following day, the slides were washed with PBS containing 0.1% Triton X-100 and incubated with secondary monoclonal or polyclonal antibodies conjugated to Alexa Fluor 488 or 568 (Dako, Carpinteria, CA, USA, 1:100) for 60 min in a humid chamber. Subsequently, the slides were washed with PBS containing 0.1% Triton X-100 and mounted with Vectashield to prevent depletion of fluorescence (Vector Laboratories, Inc., Burlingame, CA, USA) and sealed with enamel.

### 5.4. In Vitro Cytotoxicity Assay

The D17 and MG63 OSA and COBS cell lines were plated in 96-well plates in 150 µL volumes at a concentration of 0.7 × 10^4^ cells/mL for 24 h before incubation with the reengineered anthrax toxin. We used the reengineered anthrax toxin in the following concentrations: LF (500 ng/mL) + PA (0–5000 ng/mL), LF (500 ng/mL) + PA-U2-R200A + PA-L1-l210 (0–5000 ng/mL), FP59 (100 ng/mL) + PA (0–5000 ng/mL), and FP59 (100 ng/mL) + PA-U2-R200A+PA-L1-l210 (0–5000 ng/mL).

Cell viability was assessed using the MTT assay based on the metabolism of tetrazolium (3-)4,5-dymethylthiazol-2-(yl)-2,5-(diphenyl tetrazolium bromide) salts by the mitochondria of metabolically active cells. The cells were treated with concentrations ranging from 1 ng to 1 mg. After 24 h of treatment, 10 µL/well of MTT (5 mg/mL) was added to 100 µL of medium. The plate was incubated in an incubator at 5% CO_2_ and 37 °C for 3 h. Then, the plates were centrifuged and 100 µL of HCl solution (0.04 N) in isopropanol was added to each well, followed by slow pipetting to dissolve the crystals. After 10 min, the optical density was read using a spectrophotometer (Multi Skan Thermofisher Scientific, Waltham, MA, USA) at a wavelength of 570 nm [18,39,40].

### 5.5. Cell Cycle Analysis

The cell cycle analysis was performed using propidium iodide (PI) and flow cytometry. For flow cytometry, 10^6^ cells of each cell line were permeabilised by fixation in ethanol (Merck^®^, Darmstadt, Germany) or up to 3 days (−20 °C). Then, the cells were washed three times with PBS (5 min, 1200 RPM) and incubated with 200 µL of a solution containing 20 µg/mL IP, 200 µg/mL RNase A, and 0.1% Triton X-100 in PBS. After 15 min of incubation in the dark, 10,000 events were acquired by flow cytometry on the FACSCalibur device (Becton Dickinson, NJ, USA) using the program CellQuestPro. The analysis of fluorescence data and cell percentages in each phase of the cell cycle was performed using Flowjo (Tree Star Inc., Asland, OR, USA) version 10 and Flowjo version 7.6.1 (Tree Star Inc.). The results were expressed as a percentage of cells in the G1, G0/G1, S, and G2/M phases. The data were represented as histograms.

### 5.6. Apoptosis and Necrosis Assay

The apoptosis rate was determined using flow cytometry of cells labelled with annexin V and PI. A commercial kit containing Alexa^®^ Fluor 488 annexin V (Molecular Probes, Eugene, OR, USA) and PI was used to stain the cells. The kit contained the following components: A: Alexa Fluor^®^ 488 annexin V in 25 mM HEPES solution, 140 mM NaCl, 1 mM EDTA, pH 7.4, and 0.1% bovine serum albumin; B: PI at 1 mg/mL (1.5 mM) in deionised water; and C: 5× annexin binding buffer containing 50 mM HEPES, 700 mM NaCl, and 12.5 mM CaCl_2_. Component C was diluted in deionised water at a ratio of 1:4 to make the buffer. Component B was diluted in the buffer at a ratio of 1:9, yielding a final PI concentration of 100 µg/mL. The cell labelling solution was prepared by adding 2 µL of component A and 1 µL of PI for each 100 µL of buffer.

The OSA cell lines were cultured in 30 mm dishes (2 × 10^5^ cells) for 24 h. After treatment with the reengineered anthrax toxin, the culture medium was removed and the cells were trypsinised and collected in a tube for flow cytometry. After centrifugation, the cells were resuspended in 100 µL of the annexin/PI solution and incubated for 15 min at room temperature. Cells were sorted on the FACSCalibur^®^ cytometer (Becton Dickinson Immunocytometry System™, San Diego, CA, USA) by measuring the fluorescence emission at 530 and 575 nm with excitation at 488 nm, with at least 10,000 events being observed. The FlowJo^®^ software (Ashland, OR, USA) was used to analyse the data.

### 5.7. Wound-Healing Assay

The OSA cell lines were cultured in 12-well plates. After reaching 100% confluence, we used a 200 µL tip to create a “scratch” in the monolayer. Then, the culture medium and cellular debris were aspirated. New culture medium was added slowly. A photo was taken at this point to record the initial scratch width (time zero), and cells were treated with either the toxin or control. The plate was placed in an incubator at 37 °C and 5% CO_2_. After 24 h, another photo was taken under an inverted microscope to assay the wound width. To determine the migration rate, we photographed three fields and measured the total area of the wound in the monolayer in relation to the size of the field at baseline (0 h) and compared the area of the wound in the monolayer in relation to the area of the field after 24 h of treatment. We averaged the three fields and the results were presented as a percentage.

### 5.8. Statistical Analyses

GraphPad Prism 8 was used to make the graphs and perform the statistical analysis. One-way analysis of variance followed by the Dunnett post-test was used to analyse differences between different treatments. Values were considered statistically significant at *p* ≤ 0.05.

## Figures and Tables

**Table 1:** IC50 values upon treatment of D17, MG63, and COBS with the toxin parts. The IC50 values were calculated with GraphPad Prism software.

IC50	LF + PA (ng/mL)	LF + PAL1/PAU2 (ng/mL)	FP + PA (ng/mL)	FP + PAL1/PAU2 (ng/mL)
**D17**	511.2	9277	1.073	1.077
**MG63**	20,439	2070	1.057	1.082
**COBS**	3418	79,655	37.76	42.12

**Table 2:** Antibodies used for the immunofluorescence analysis of canine and human OSA and COBS cells.

Antibody	Catalogue Number	Mono or Polyclonal	Mouse or Rabbit	Dilution	Subcellular Localisation
*MMP-2–Abcam*	Ab86607	Monoclonal	Mouse	1:200	Membrane
uPA H140–Santa Cruz	Sc14019	Polyclonal	Rabbit	1:200	Cytoplasm
β-catenin-Invitrogen	138400	Monoclonal	Mouse	1:100	Membrane, cytoplasm, and nucleus
p53-Serotec	MCA909	Monoclonal	Mouse	1:100	Nucleus
MAPK-Cell Signaling	2532	Polyclonal	Rabbit	1:100	Cytoplasm

## References

[1] Morello E., Martano M., Buracco P. (2011). Biology, diagnosis and treatment of canine appendicular osteosarcoma: Similarities and differences with human osteosarcoma. Vet. J..

[2] Withrow S.J., Powers B.E., Straw R.C., Wilkins R.M. (1991). Comparative aspects of osteosarcoma. Dog versus man. Clin. Orthop. Relat. Res..

[3] Misdorp W., Van der Heul R.O. (1976). Tumours of bones and joints. Bull. World Health Organ..

[4] Covey J.L., Farese J.P., Bacon N.J., Schallberger S.P., Amsellem P., Cavanaugh R.P., Milner R.J. (2014). Stereotactic radiosurgery and fracture fixation in 6 dogs with appendicular osteosarcoma. Vet. Surg..

[5] Selvarajah G.T., Bonestroo F.A., Kirpensteijn J., Kik M.J., van der Zee R., van Eden W., Timmermans-Sprang E.P., Slob A., Mol J.A. (2013). Heat shock protein expression analysis in canine osteosarcoma reveals HSP60 as a potentially relevant therapeutic target. Cell Stress Chaperones.

[6] Farese J.P., Kirpensteijn J., Kik M., Bacon N.J., Waltman S.S., Seguin B., Kent M., Liptak J., Straw R., Chang M.N. (2009). Biologic behavior and clinical outcome of 25 dogs with canine appendicular chondrosarcoma treated by amputation: A Veterinary Society of Surgical Oncology retrospective study. Vet. Surg..

[7] Casteleyn C., Sleeckx N., De Spiegelaere W., Heindryckx F., Coulon S., Van Steenkiste C. (2013). New therapeutic targets in veterinary oncology: Man and dog definitely are best friends. Vet. J..

[8] Castro J.L.C., Santalucia S., Nazareth W., Castro V.S.P., Pires M.V.M., Leme P.T.O., Paula L.R., Ururahy K.C.B., Corrêa L.F.D., Raiser A.G. (2013). Axial osteosarcoma in dog—Case report. J. Vet. Adv..

[9] Kirpensteijn J., Kik M., Rutteman G.R., Teske E. (2002). Prognostic significance of a new histologic grading system for canine osteosarcoma. Vet. Pathol..

[10] Selvarajah G.T., Kirpensteijn J. (2010). Prognostic and predictive biomarkers of canine osteosarcoma. Vet. J..

[11] Bachran C., Leppla S.H. (2016). Tumor targeting and drug delivery by anthrax toxin. Toxins.

[12] Liu S., Aaronson H., Mitola D.J., Leppla S.H., Bugge T.H. (2003). Potent antitumor activity of a urokinase-activated engineered anthrax toxin. Proc. Natl. Acad. Sci. USA.

[13] Liu S., Bugge T.H., Leppla S.H. (2001). Targeting of tumor cells by cell surface urokinase plasminogen activator-dependent anthrax toxin. J. Biol. Chem..

[14] Liu S., Bugge T.H., Frankel A.E., Leppla S.H. (2009). Dissecting the urokinase activation pathway using urokinase-activated anthrax toxin. Methods Mol. Biol..

[15] Klimpel K.R., Molloy S.S., Thomas G., Leppla S.H. (1992). Anthrax toxin protective antigen is activated by a cell surface protease with the sequence specificity and catalytic properties of furin. Proc. Natl. Acad. Sci. USA.

[16] Liu S., Redeye V., Kuremsky J.G., Kuhnen M., Molinolo A., Bugge T.H., Leppla S.H. (2005). Intermolecular complementation achieves high-specificity tumor targeting by anthrax toxin. Nat. Biotechnol..

[17] Rønø B., Rømer J., Liu S., Bugge T.H., Leppla S.H., Kristjansen P.E. (2006). Antitumor efficacy of a urokinase activation-dependent anthrax toxin. Mol. Cancer Ther..

[18] Schafer J.M., Peters D.E., Morley T., Liu S., Molinolo A.A., Leppla S.H., Bugge T.H. (2011). Efficient targeting of head and neck squamous cell carcinoma by systemic administration of a dual uPA and MMP-activated engineered anthrax toxin. PLoS ONE.

[19] Nishiya A.T., Nagamine M.K., Fonseca I.I.M.D., Miraldo A.C., Scattone N.V., Guerra J.L., Xavier J.G., Santos M., Gomes C.O.M.S., Ward J.M. (2020). Inhibitory Effects of a reengineered anthrax toxin on canine oral mucosal melanomas. Toxins.

[20] Andreasen P.A., Egelund R., Petersen H.H. (2000). The plasminogen activation system in tumor growth, invasion, and metastasis. Cell. Mol. Life Sci..

[21] Fonseca J.M. (2019). Effects of *Bacillus anthracis* Reengineered Toxin (PA-U2-R200A + PA-L1-I210A + LF) on Canine Osteosarcoma. Ph.D. Thesis.

[22] Liu S., Liu J., Ma Q., Cao L., Fattah R.J., Yu Z., Bugge T.H., Finkel T., Leppla S.H. (2016). Solid tumor therapy by selectively targeting stromal endothelial cells. Proc. Natl. Acad. Sci. USA.

[23] Ezzell J.W., Ivins B.E., Leppla S.H. (1984). Immunoelectrophoretic analysis, toxicity, and kinetics of in vitro production of the protective antigen and lethal factor components of *Bacillus anthracis* toxin. Infect. Immun..

[24] Wein A.N., Peters D.E., Valivullah Z., Hoover B.J., Tatineni A., Ma Q., Fattah R., Bugge T.H., Leppla S.H., Liu S. (2015). An anthrax toxin variant with an improved activity in tumor targeting. Sci. Rep..

[25] Liu S., Wang H., Currie B.M., Molinolo A., Leung H.J., Moayeri M., Basile J.R., Alfano R.W., Gutkind J.S., Frankel A.E. (2008). Matrix metalloproteinase-activated anthrax lethal toxin demonstrates high potency in targeting tumor vasculature. J. Biol. Chem..

[26] Duesbery N.S., Webb C.P., Leppla S.H., Gordon V.M., Klimpel K.R., Copeland T.D., Ahn N.G., Oskarsson M.K., Fukasawa K., Paull K.D. (1998). Proteolytic inactivation of MAP-kinase-kinase by anthrax lethal factor. Science.

[27] Moayeri M., Leppla S.H., Vrentas C., Pomerantsev A.P., Liu S. (2015). Anthrax Pathogenesis. Annu. Rev. Microbiol..

[28] Abi-Habib R.J., Singh R., Leppla S.H., Greene J.J., Ding Y., Berghuis B., Duesbery N.S., Frankel A.E. (2006). Systemic anthrax lethal toxin therapy produces regressions of subcutaneous human melanoma tumors in athymic nude mice. Clin. Cancer Res..

[29] Su Y., Ortiz J., Liu S., Bugge T.H., Singh R., Leppla S.H., Frankel A.E. (2007). Systematic urokinase-activated anthrax toxin therapy produces regressions of subcutaneous human non-small cell lung tumor in athymic nude mice. Cancer Res..

[30] Liu S., Netzel-Arnett S., Birkedal-Hansen H., Leppla S.H. (2000). Tumor cell-selective cytotoxicity of matrix metalloproteinase-activated anthrax toxin. Cancer Res..

[31] Santos A., Lopes C., Marques R.M., Amorim I., Ribeiro J., Frias C., Vicente C., Gartner F., de Matos A. (2011). Immunohistochemical analysis of urokinase plasminogen activator and its prognostic value in canine mammary tumours. Vet. J..

[32] Anwar S., Yanai T., Sakai H. (2015). Immunohistochemical Detection of Urokinase Plasminogen Activator and Urokinase Plasminogen Activator Receptor in Canine Vascular Endothelial Tumours. J. Comp. Pathol..

[33] Rossmeisl J.H., Hall-Manning K., Robertson J.L., King J.N., Davalos R.V., Debinski W., Elankumaran S. (2017). Expression and activity of the urokinase plasminogen activator system in canine primary brain tumors. OncoTargets Ther..

[34] Ramos S.C., de Matos A.J., Ribeiro J.N., Leite-Martins L.R., Ferreira R.R.F., Viegas I., Santos A.A. (2017). Serum levels of urokinase-type plasminogen activator in healthy dogs and oncologic canine patients. Vet. World.

[35] McGill L.D. (2011). Human and canine mammary tumors: A role for urokinase plasminogen activator?. Vet. J..

[36] Hoekstra R., Eskens F.A., Verweij J. (2001). Matrix metalloproteinase inhibitors: Current developments and future perspectives. Oncologist.

[37] Levinsohn J.L., Newman Z.L., Hellmich K.A., Fattah R., Getz M.A., Liu S., Sastalla I., Leppla S.H., Moayeri M. (2012). Anthrax lethal factor cleavage of Nlrp1 is required for activation of the inflammasome. PLoS Pathog..

[38] Mueller F., Fuchs B., Kaser-Hotz B. (2007). Comparative biology of human and canine osteosarcoma. Anticancer Res..

[39] Simpson S., Dunning M.D., de Brot S., Grau-Roma L., Mongan N.P., Rutland C.S. (2017). Comparative review of human and canine osteosarcoma: Morphology, epidemiology, prognosis, treatment and genetics. Acta Vet. Scand..

[40] Gustafson D.L., Duval D.L., Regan D.P., Thamm D.H. (2018). Canine sarcomas as a surrogate for the human disease. Pharmacol. Ther..

[41] Fenger J.M., London C.A., Kisseberth W.C. (2014). Canine osteosarcoma: A naturally occurring disease to inform pediatric oncology. ILAR J..

